# Electrocautery therapy combined with oral steroid administration for refractory corrosive esophageal stenosis prevents restenosis

**DOI:** 10.1007/s10388-013-0375-7

**Published:** 2013-04-20

**Authors:** Kouichi Nonaka, Shinichi Ban, Masayasu Aikawa, Akira Yamasaki, Ayako Okuda, Takeyasu Kounoe, Hideaki Naoe, Kouichi Sakurai, Mitsuo Miyazawa, Hiroto Kita, Yutaka Sasaki

**Affiliations:** 1 Department of Gastroenterology and Hepatology, Graduate School of Medical Sciences, Kumamoto University, Kumamoto, 860-0856 Japan; 2 Department of Pathology, Saiseikai Kawaguchi General Hospital, Kawaguchi, 332-8558 Japan; 3 Department of Surgery, Saitama Medical University International Medical Center, Hidaka, 350-1298 Japan; 4 Department of Gastroenterology, Saitama Medical University International Medical Center, Hidaka, 350-1298 Japan

**Keywords:** Esophageal stenosis, Steroid, Dilation

## Abstract

A 61-year-old female with refractory corrosive esophageal stenosis repeatedly underwent endoscopic balloon dilation at another hospital; however, no improvements were observed in the esophageal stenosis. Consequently, she had been on a liquid diet for the previous three years. She was admitted to our department for further treatment. A radial incision was made, by use of the SB knife Jr, for a pinhole-like stenosis in a short segment 39 cm from the incisor, and dilation was safely performed by use of a CRE balloon dilator. Subsequently, prednisolone was orally administered to prevent re-stenosis. This was followed by a favorable clinical course.

## Introduction

Refractory benign esophageal stenosis makes it difficult to consume meals, which markedly reduces a patient’s quality of life. Endoscopic balloon dilation (EBD) is commonly used for treatment of this condition. However, dilation can cause esophageal perforation in some patients, and sufficient dilation cannot be achieved for others with refractory stenosis, despite repeated EBDs. Moreover, patient management can be very difficult. In this study, to treat a patient with refractory corrosive esophageal stenosis safely and effectively, we used an endoscopic submucosal dissection (ESD) device, the SB knife Jr, combined with oral steroid therapy, which is reportedly useful for preventing restenosis after dilation for esophageal stenosis.

## Case

Esophageal stenosis developed in a 61-year-old female approximately 4 years after she had attempted suicide by taking a sulfate. She had no history of underlying diseases including mental diseases. She attempted suicide during a domestic quarrel. Although the patient had frequently undergone balloon dilation at another hospital, no improvements were observed in her condition. The patient was referred to the Department of Surgery in our hospital, and underwent further dilation. After the procedure, the esophagus was observed to be perforated, with hematoma formation being noted around the esophagus. When laparotomy was performed to remove the hematoma, the stenosis became exacerbated. After 3 months, she was referred and admitted to our department for further treatment. Although she had been on a liquid diet for the previous 3 years, no abnormalities were found in her hematological data. Her height, body weight, and body mass index (BMI) were 158.4 cm, 58.6 kg, and 23.4, respectively. Malnutrition was not noted.

**Fig. 1:**
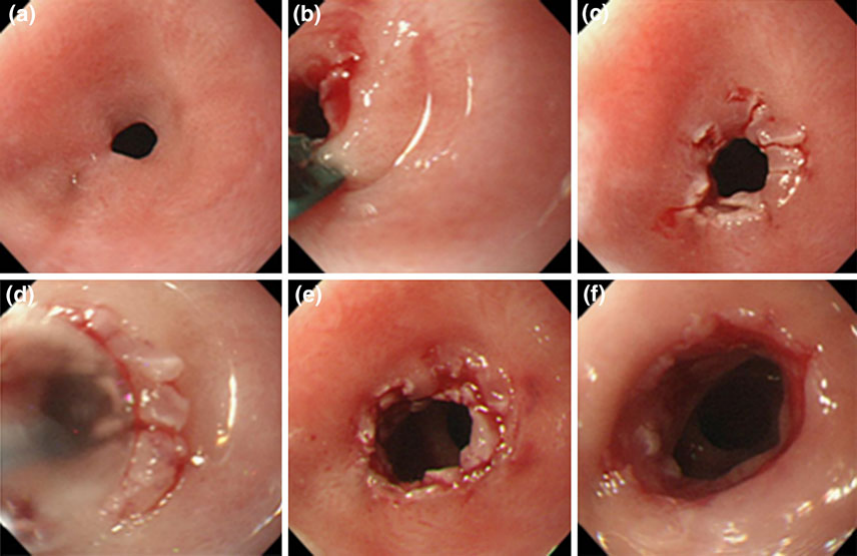
Electrocautery therapy in this case. **a** Endoscopic findings of marked esophageal stenosis before treatment. A pinhole-like circumferential membranous stenosis was observed in an area 39 cm from the incisor. An endoscope could not be passed through this area. **b** Incision of the membranous region of the stenotic site by use of the SB knife Jr. **c** Endoscopic findings after the circumferential incision. **d** Endoscopic findings after balloon dilation after the incision. **e** Endoscopic findings after completion of the first endoscopic dilation. A GIF-XP240 endoscope (maximum diameter 7.7 mm) was successfully passed through the opening. **f** Endoscopic findings after additional incision and balloon dilation after 2 days. A GIF-Q260 endoscope (maximum diameter 9.2 mm) was successfully passed through the opening

After she had been admitted, upper digestive tract endoscopy using a GIF-XQ260 endoscope (Olympus Optical, Tokyo, Japan) with an end diameter of 9.0 mm was performed to confirm the stenotic site. A pinhole-like stenosis was observed in an area 39 cm from the incisor, which made it impossible to pass the endoscope further into the tract (Fig. 1a). When dilating the stenosis with the dilation procedure (CRE balloon dilator; Boston Scientific, Boston, MA, USA; 6–8 mm) under fluoroscopy, the stenotic site was found to be relatively soft, indicative of a short-segment stenosis.

Subsequently, stenosis-reducing surgery was performed by use of a GIF-XQ260 endoscope. An ESD device, the SB knife Jr (MD-47703; Sumitomo Bakelite, Tokyo, Japan), was used to make an incision in the stenotic site. During this procedure, the membranous stenotic site was initially captured by use of the SB knife Jr, with an incision being performed while drawing the tissue in Endocut mode set at effect 3 (output 80 W) (Fig. 1b). The membranous stenotic site was then incised as radially as possible (Fig. 1c). Dilation was performed by use of a CRE balloon dilator (Boston Scientific; 8–10 mm), with inflation continuing until the diameter reached 10 mm (Fig. 1d, e).

Next day, endoscopy revealed re-stenosis. A second dilation was therefore performed, with the balloon being inflated to a diameter of 12 mm, which made it possible to pass a GIF-Q260 endoscope (Olympus Optical) with an end diameter of 9.2 mm through the tract (Fig. 1f).

**Fig. 2:**
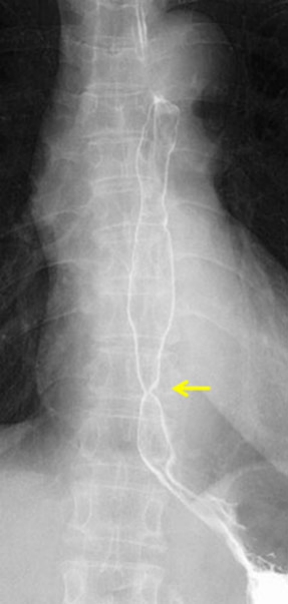
Fluoroscopy before electrocautery therapy in this case. As observed endoscopically, a tight stenosis was noted in the lower thoracic esophagus (*arrow*). The major axis of the stenosis was less than 10 mm, and it was a short-segment stenosis

Fluoroscopy findings before electrocautery therapy are shown in Fig. 2. In accordance with the endoscopic findings, severe stenosis was observed in the lower thoracic esophagus. There was no stenosis in the upper and middle thoracic esophagus. The major axis of the stenosis was less than 10 mm, and it was a short-segment stenosis.

**Fig. 3:**
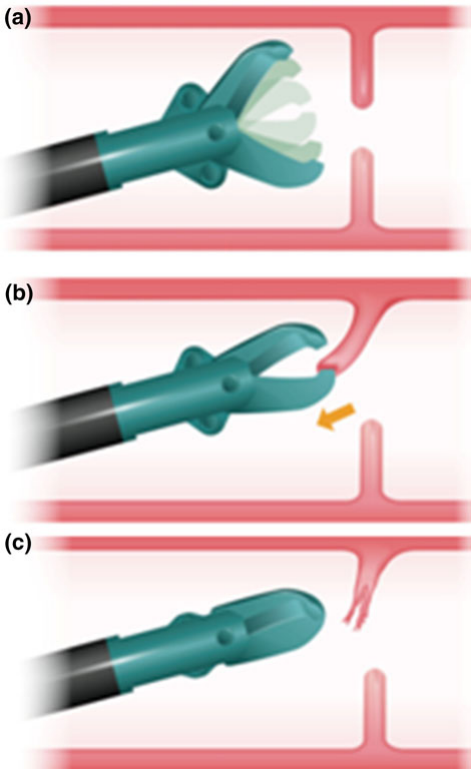
Schematic diagram of electrocautery therapy by use of the SB knife Jr. On approaching the membranous stenosis, the SB knife Jr was opened fully (**a**), followed by tight capture and drawing in of the target tissue (**b**). Finally, the membranous site was rapidly incised (**c**)

The procedure used for stenosis-reducing surgery is illustrated schematically in Fig. 3; after approaching the membranous stenosis, the SB knife Jr was opened fully (Fig. 3a), followed by tight capture (Fig. 3b). Finally, the membranous site was rapidly incised (Fig. 3c).

**Fig. 4:**
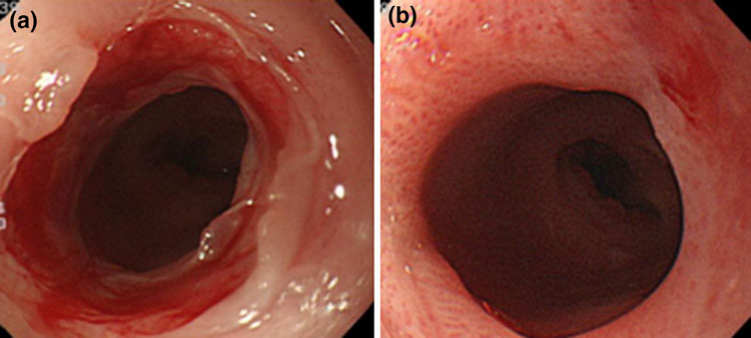
Clinical course after electrocautery therapy. **a** Endoscopy findings after oral administration of prednisolone (20 mg/day) for 2 weeks. Although there was no tendency toward stenosis, prophylactic dilation was performed. **b** Endoscopy findings 3 months after the first treatment. The mucosa at the stenotic site where the treatment was performed was completely covered by regenerated epithelium. A GIF-Q260 endoscope was successfully passed through the opening and no restenosis was observed

Dietary ingestion was started next day, in conjunction with oral administration of prednisolone at 20 mg/day for 2 weeks. Subsequently, the dose was reduced by 5 mg/week over a total administration period of 5 weeks. After initiation of the prednisolone therapy, follow-up endoscopy was performed at one-week intervals. To prevent restenosis, further 12 mm diameter dilations were conducted by use of a CRE balloon dilator 1, 2 (Fig. 4a), 3, and 4 weeks after prednisolone administration was started (4 times in total). The patient was discharged on the day after the second balloon dilation, and she underwent the 3rd and 4th balloon dilations and steroid dose reduction at the outpatient clinic. On completion of prednisolone therapy, it became possible to pass a GIF-Q260 endoscope through the tract without restrictions (Fig. 4b). The dilation site was covered by regenerated epithelium. No restenosis was observed during the 10-month follow-up. Dietary restrictions became unnecessary, and the patient’s body weight has increased by 5 kg.

## Discussion

Endoscopic dilation is usually the initial treatment for benign esophageal stenosis [1]. However, because not all cases respond to this treatment, surgery may be required for some patients. Recent studies have described the usefulness of self-expanding removable and biodegradable stents [2, 3]. Although these treatments have been performed successfully, limitations are associated with their use, for example migration and epithelial hyperplasia-related stenosis [4, 5].

ESD techniques with needles and IT knives have been shown to be useful for relieving esophageal stenosis/anastomotic-site stenosis [6–8]. However, use of these devices may be limited, because of perforations [8]. The IT knife, also used by Muto et al. in their report [8], is equipped with a ball chip made of an insulating material at the tip and is capable of safer incision of stenotic regions than needle knives. However, experience is needed to successfully attach the knife to the stenotic site to be incised and adjust the strength of outward tension. Adjusting the size of the incision is also slightly difficult. Because of these problems, the incidence of perforations was 3.5 % in the report by Muto et al.

This study was therefore designed to investigate stenosis-reducing surgery with a scissor-type ESD device, for safer and more accurate relief of a stenosis than with an IT knife. Use of the ESD scissor-type knife, the SB knife Jr, makes it possible to safely perform incisions while capturing and drawing the target tissue inward. Because it was easy to capture and cut the tissue, we were able to use the device to successfully treat the stenosis of this particular patient. The additional benefits of using this knife include being able to capture the target tissue several times, and being able to precisely control the tissue volume to be incised by regulating the volume of tissue captured. Furthermore, circumferential and uniform incisions can be performed by use of this method. This makes it possible to safely conduct balloon dilation without adding pressure to any single point. A disadvantage is that the indication is only short-segment stenosis.

In this case, repeated endoscopic dilation over several years resulted in a perforation during the dilation procedure. However, favorable dilation was ultimately achieved by combined use of an electrocautery incision with the SB knife Jr, and oral administration of a steroid, which helped prevent restenosis after the stenosis-reducing surgery.

Several previous studies have described the effectiveness of topically injected or orally administered steroids for prevention of restenosis after balloon dilation for esophageal stenosis or post-ESD stenosis [9]. However, the exact mechanism has yet to be clarified. In this regard, we performed a preliminary animal experiment in which a steroid was administered after circumferential esophageal ESD. Esophageal circumferential mucosal defects were created in four pigs by endoscopic mucosal dissection (ESD). One pig was sacrificed five minutes after ESD, and another two pigs were followed-up by endoscopy and sacrificed 1 and 3 weeks after ESD. The remaining pig was followed-up by endoscopy, received a local steroid injection five times, and was sacrificed 8 weeks after ESD. The esophageal tissues of all the pigs were subjected to pathological analysis.

Consequently, the number of myofibroblasts appearing in the healing process was lower than that in the steroid-untreated group and their morphology and arrangement was different (unpublished data). Because myofibroblasts have been reported to have the ability to contract [10], we speculate that steroid treatment may prevent stenosis by changing the amount, morphology, and arrangement of myofibroblasts. Further analysis is needed to clarify the role of steroid treatment.

In conclusion, we encountered a case of refractory benign esophageal stenosis that required frequent dilation over a long period of time. Our case demonstrated that dilation using our newly developed ESD technique in conjunction with steroid therapy resulted in a favorable clinical course.

The method reported herein may be suitable for treatment of short-segment stenosis, and may potentially be useful as an novel treatment for refractory benign esophageal stenosis, especially membranous stenosis. Moreover, it may serve as a beneficial treatment for postoperative anastomotic-site stenosis and congenital membranous stenosis.
